# Combined Exposure to Deoxynivalenol and Patulin Aggravates Liver Injury in Mice via Triggering Inflammation, Apoptosis, and Oxidative Stress

**DOI:** 10.3390/toxins18080355

**Published:** 2026-08-20

**Authors:** Qingqing Zhao, Zenghao Xu, Xianglong Dai, Xingyu Zhang, Maolong Li, Juan Chang, Qingqiang Yin, Guoyu Yang, Chaoqi Liu

**Affiliations:** 1 College of Veterinary Medicine, Henan Agricultural University, Zhengzhou 450046, China; zqq900924@163.com (Q.Z.); xuzenghaocr7@163.com (Z.X.); haubiochem@163.com (G.Y.); 2 College of Animal Science and Technology, Henan Agricultural University, Zhengzhou 450046, China; 17630732025@163.com (X.D.); zhangxingyu0325@163.com (X.Z.); changjuan2000@126.com (J.C.); qqy1964@henau.edu.cn (Q.Y.); 3 Henan Delin Biological Product Co., Ltd., Xinxiang 453000, China; limaolong2012@163.com

**Keywords:** DON, PAT, mice, inflammation, apoptosis, oxidative stress

## Abstract

Deoxynivalenol (DON) and patulin (PAT) are common mycotoxins in cereals and fruits, posing health risks for animals and human beings. In order to study their liver toxicity, 24 mice were randomly assigned to four groups, with six replicates in each group (one mouse per cage). The mice were intragastrically administered with DON, PAT, DON + PAT (DP), or without DON and PAT (the control group) for 28 days, respectively. The results showed that body weight gain was significantly reduced by all toxin treatments, compared with the control group, and the lowest body weight gain was observed in the DP group. Histopathology revealed that hepatocyte damage and inflammatory infiltration were more serious in the DP group, exhibiting the highest mRNA abundances of MyD88, IFN-γ, and JAK2. The severity of hepatocyte apoptosis induced in each group followed the order: DON > DP > PAT; the severity of oxidative stress was ranked as DP > DON > PAT. Transcriptomic analysis revealed that numerous differentially expressed genes were regulated by DP treatment, which were mainly enriched in the MAPK, JAK-STAT, and transforming growth factor (TGF)-β signaling pathways. In conclusion, individual exposure to DON or PAT triggered hepatic injury, and their co-exposure further exacerbated liver damage.

## 1. Introduction

Mycotoxins are toxic secondary metabolites produced by fungi including *Aspergillus*, *Fusarium*, and *Penicillium*. They are characterized by high stability and widely contaminate and accumulate in cereals and various foods; however, their presence is often inconspicuous [1]. Food quality is impaired, and persistent and severe threats to human and animal health are posed through multiple processes including immunosuppression and organ damage [2]. Over 400 mycotoxin species have been isolated and identified [3]. Among these, deoxynivalenol (DON) and patulin (PAT) are regarded as two representative and widely distributed mycotoxins in human and animal food products [4]. DON is mainly produced by fungi including *Fusarium graminearum*. It is widely detected as a contaminant in cereals and corresponding processed products, such as wheat, maize, and barley, etc. A detection rate of DON higher than 70% has been observed in cereals globally, while rates exceeding the regulatory limits have been documented to range from 20.4% to 21.5% in Northern and Central Europe [5]. Furthermore, the risk of contamination with more than one mycotoxin is considerably elevated in years with heavy rainfall. PAT is metabolically produced by toxigenic fungi including *Penicillium* and *Aspergillus* species. It is a hallmark contaminant in fruits such as apples, pears, grapes, and their processed products, which cannot be completely eliminated during food processing [6,7,8]. PAT has also been detected in cereals, legumes, seeds, nuts, and vegetables [9]. Nevertheless, insufficient attention has been paid to PAT. Clear regulatory limits have been established only for fruits and their processed products; however, PAT has not received the same extensive attention as DON [10,11]. The co-occurrence of multiple mycotoxins, including dual contamination, is widely prevalent in food and feedstuffs [12,13]. Additive toxic effects have been well demonstrated when certain mycotoxins, such as aflatoxin B_1_ (AFB_1_) and DON, coexist in matrices [13]. Notably, additive toxic effects have been well demonstrated when certain mycotoxins such as aflatoxin B1 (AFB1) and DON coexist in matrices [14]. Accordingly, whether additive toxic effects can be triggered by the combined presence of DON and PAT remains to be elucidated.

As the main metabolic and detoxifying organ in organisms, the liver is easily damaged by mycotoxins. Thus, hepatotoxicity serves as a critical endpoint in the risk assessment of such pollutants [15]. DON and PAT, as common food contaminants, have been confirmed to exert significant hepatotoxicity individually. Intragastric administration of 2.4 mg/kg body weight of DON to mice for 28 days and intragastric administration of 4 mg/kg body weight of PAT induced pathological changes in liver tissues [16,17]. Additionally, excessive oxidative stress can be induced in hepatic tissue by DON or PAT exposure, accompanied by the massive generation of reactive oxygen species, decreased antioxidant capacity of the body, and elevated lipid peroxidation levels [18,19,20]. Furthermore, persistent inflammatory responses in the liver are activated, and the release of pro-inflammatory factors is upregulated, which further triggers hepatocyte apoptosis and liver tissue lesions [21,22,23]. Only one report was found concerning the combined hepatocellular carcinoma toxicity of DON and PAT [24]. Whether the combination of these two toxins exhibits stronger hepatic toxicity in animals than individual toxins has not been reported. Therefore, it is of great scientific significance to investigate the hepatic toxicity and molecular mechanism of action in mice treated with DON, PAT, or their combination.

## 2. Results

### 2.1. Effects of Individual or Combined Treatment with DON and PAT on Growth Performance and Hepatic Organ Index Values in Mice

**Figure 1:**
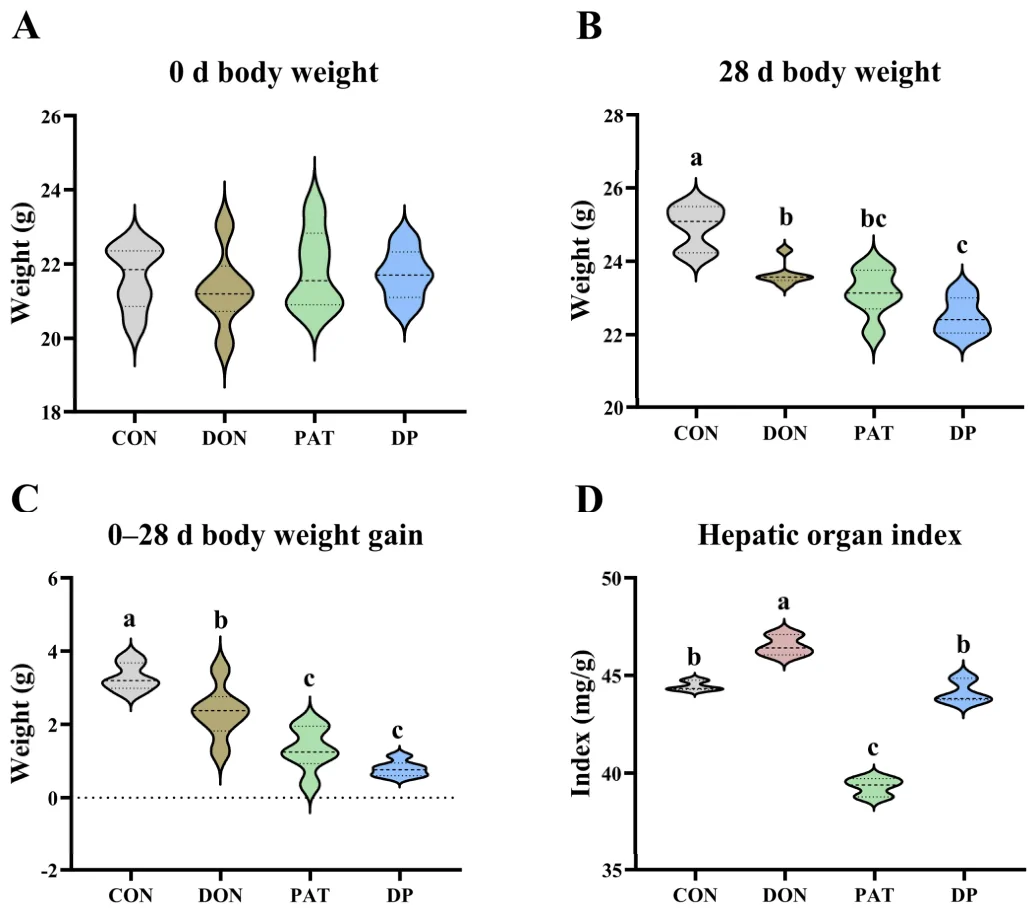
Individual and combined effects of DON and PAT on mice growth and hepatic indices (*n* = 6). Note: CON: control group; DON: 1.5 mg/kg/d DON group; PAT: 2 mg/kg/d PAT group; DP: 1.5 mg/kg/d DON + 2 mg/kg/d PAT group. (**A**,**B**) Body weights of mice in each group at the ages of 0 and 28 days, respectively. (**C**) The body weight gain of mice during the 0–28 d period. The horizontal dashed line indicates the zero baseline for body weight gain; (**D**) Liver indexes of mice in each group. The same lowercase letters (e.g., a, b and c) as superscripts indicate statistically insignificant differences between treatment groups (*p* > 0.05), whereas the different lowercase letters denote statistically significant differences (*p* < 0.05).

No significant difference was observed in the initial body weight among all groups of 6-week-old mice, suggesting uniform grouping suitable for subsequent experiments (Figure 1A). Figure 1B showed that body weight was reduced in mice exposed to DON, PAT or DP, compared with the CON group. Specifically, the body weight of mice on the 28th day in the DON, PAT, and DP groups was significantly lower than that in the CON group (*p* < 0.05), whereas that in the DON group was significantly higher than that in the DP group (*p* < 0.05). No significant difference was found between the DON and PAT groups or PAT and DP groups (*p* > 0.05). Body weight gain in the DON, PAT, and DP groups was significantly reduced, compared with the CON group (*p* < 0.05) (Figure 1C), which was insignificantly different between the PAT and DP groups (*p* > 0.05). Figure 1D showed that there was no significant difference in the hepatic organ index values between the DP and CON groups (*p* > 0.05). However, the hepatic indices in both DP and CON groups were significantly lower than those in the DON group, and significantly higher than those in the PAT group. In conclusion, the growth of mice was inhibited by exposure to DON, PAT, or DP; hepatic index changes were observed in mice treated with DON or PAT alone.

### 2.2. Effects of Individual or Combined Exposure to DON and PAT on Hepatic Histomorphology in Mice

**Figure 2:**
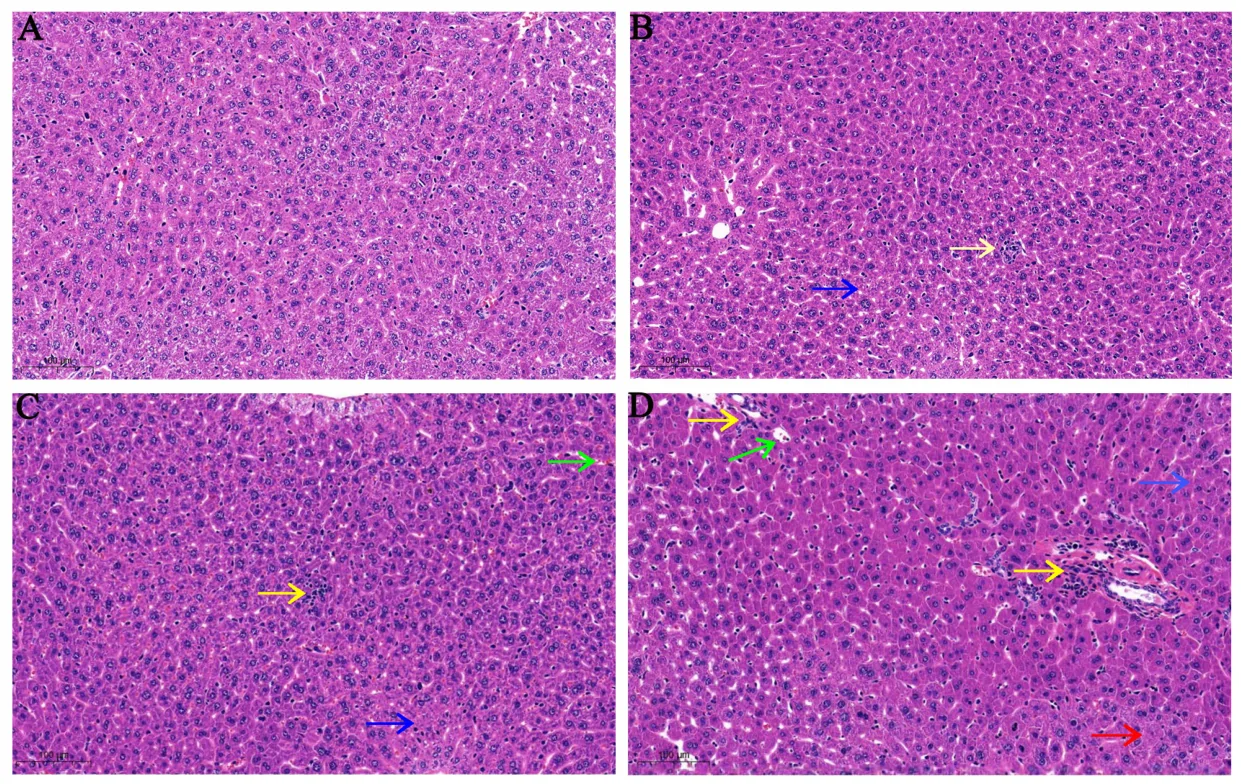
Histopathological alterations in mouse liver tissues influenced by treatment with DON, PAT, or DP (*n* = 3). Note: (**A**) control group; (**B**) 1.5 mg/kg/d DON group; (**C**) 2 mg/kg/d PAT group; (**D**) 1.5 mg/kg/d DON + 2 mg/kg/d PAT group. Liver sections were stained with hematoxylin and eosin (H&E), and the magnification was 30×. Yellow arrows: inflammatory cell infiltration; blue arrows: karyorrhexis; green arrows: hepatocyte karyolysis; red arrows: disordered hepatocyte arrangement.

Disordered hepatocyte arrangement, karyolysis, karyorrhexis, and inflammatory cell infiltration were observed in mouse liver tissues after treatment with DON, PAT, or DP, compared with the CON group (Figure 2). More severe liver tissue damage was found in the DP group, where massive inflammatory cell infiltration was detected. These results indicate that morphological damage to liver tissue was induced by a single exposure to DON or PAT. Furthermore, DON and PAT co-exposure aggravated inflammation and exacerbated hepatic damage in mice.

### 2.3. Effects of Individual or Combined Exposure to DON and PAT on Hepatic Apoptosis in Mice

**Figure 3:**
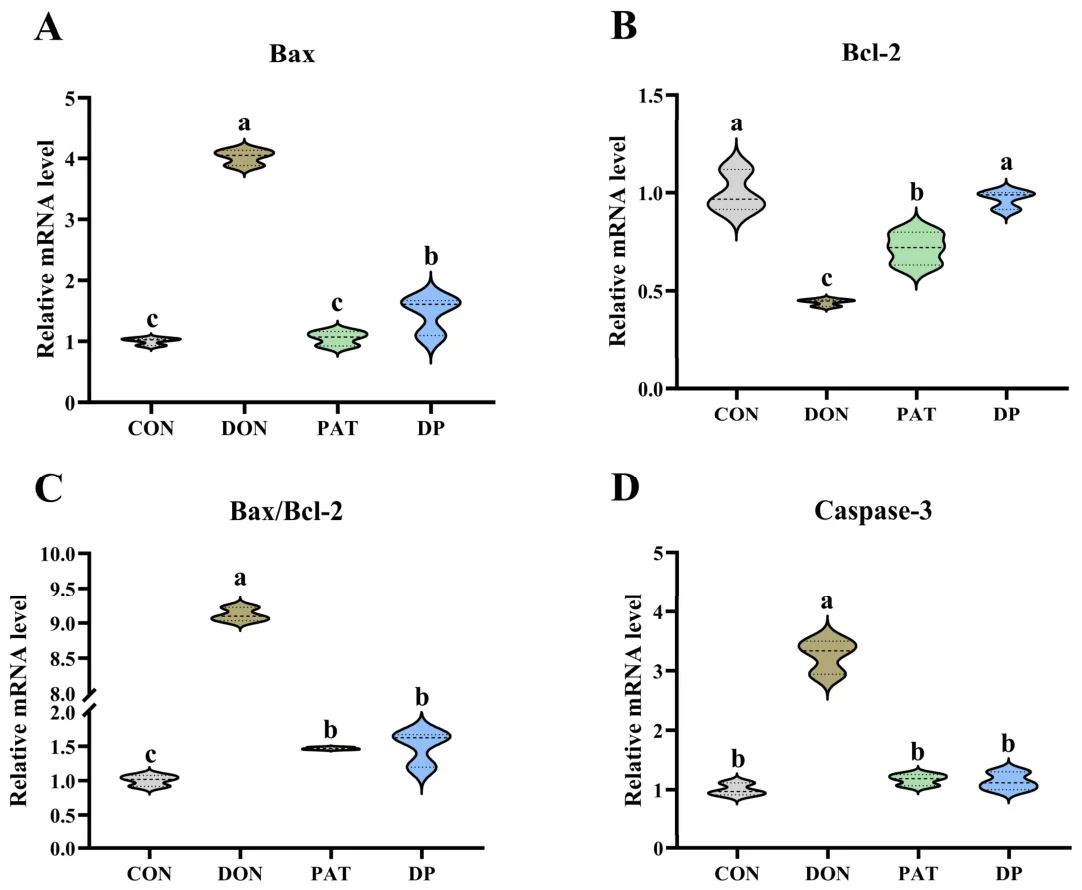
Individual and combined effects of DON and PAT on hepatic apoptosis in mice (*n* = 3). Note: (**A**–**D**) represent the mRNA abundances of Bax, Bcl-2, Bax/Bcl-2 ratio, and Caspase-3 in mouse liver, respectively. Identical lowercase superscript letters (e.g., a, b, c) indicate no significant differences among treatment groups (*p* > 0.05); different lowercase superscript letters indicate significant differences (*p* < 0.05).

The mRNA abundances of Bax and caspase-3 or the Bax/Bcl-2 ratio in the DON group were significantly higher than those in the CON, PAT, and DP groups, whereas the mRNA abundance of Bcl-2 in the DON group was significantly lower than that in the other groups (*p* < 0.05) (Figure 3). These results indicate that hepatocyte apoptosis induced by DON treatment in the mice was more severe than that from PAT treatment or combined treatment with DON and PAT. The mRNA abundance of Bax was insignificantly different between the PAT and CON groups (*p* > 0.05), which was significantly lower than that in the DP group. The mRNA abundance of Bcl-2 in CON and DP groups was significantly higher than that in the DON and PAT groups (*p* < 0.05). The ratio of Bax/Bcl-2 mRNA abundance in the CON group was the lowest, compared with other groups (*p* < 0.05), without a significant difference between the PAT and DP groups (*p* > 0.05). Additionally, Caspase-3 mRNA abundance was insignificantly different among CON, PAT, and DP groups (*p* > 0.05). These findings suggest that the hepatocyte apoptosis induced by DON in mice was the most severe, followed by co-exposure to DON and PAT (DP group), and PAT alone triggered the mildest hepatic apoptosis.

### 2.4. Effects of Individual or Combined Exposure to DON and PAT on Hepatic Inflammation in Mice

**Figure 4:**
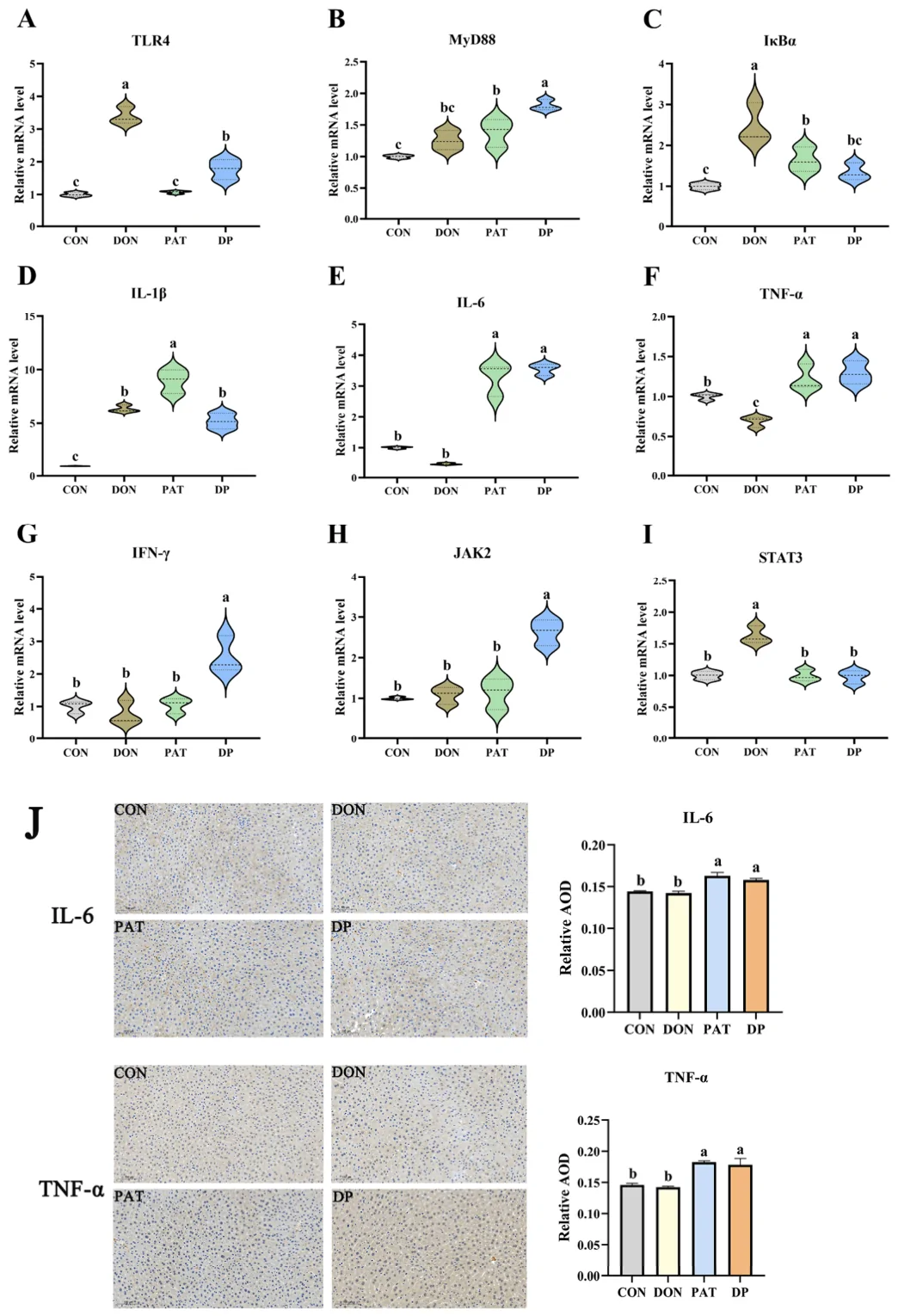
Individual and combined effects of DON and PAT on the expression of liver inflammation-related genes in mice (*n* = 3). Note: (**A**–**I**): mRNA abundances of TLR4, MyD88, IκBα, IL-1β, IL-6, TNF-α, IFN-γ, JAK2, and STAT3; (**J**) representative IHC images and relative protein expression of IL-6 and TNF-α in liver tissue. The magnification was 60×. Identical lowercase superscript letters (e.g., a, b, c) indicate no significant differences among treatment groups (*p* > 0.05); different lowercase superscript letters indicate significant differences (*p* < 0.05).

The mRNA abundances of TLR4, IκBα, IL-1β, and STAT3 were significantly upregulated in the DON group, compared with the CON group (*p* < 0.05) (Figure 4A–I). No significant alterations were detected in the mRNA abundances of MyD88, IL-6, IFN-γ, or JAK2 (*p* > 0.05), whereas the mRNA abundance of TNF-α in the DON group was significantly reduced (*p* < 0.05). The mRNA levels of MyD88, IκBα, IL-1β, IL-6, and TNF-α were significantly elevated in the PAT group, compared with the CON group (*p* < 0.05), while no significant differences were observed in the mRNA levels of TLR4, IFN-γ, JAK2 or STAT3 between both groups (*p* > 0.05). In the DP group, the mRNA levels of TLR4, MyD88, IL-1β, IL-6, TNF-α, IFN-γ and JAK2 were markedly higher than those in the CON group (*p* < 0.05); only the mRNA levels of IκBα and STAT3 showed no significant changes (*p* > 0.05). The distribution and relative average optical density (AOD) of IL-6 and TNF-α in liver tissues were displayed in Figure 4J. No significant differences were found in the numbers of IL-6-positive and TNF-α-positive cells in the DON group, compared with the CON group (*p* > 0.05). However, the numbers of IL-6-positive and TNF-α-positive cells in the PAT and the DP groups were significantly higher than those in the CON and DON groups (*p* < 0.05), which were consistent with the changes in TNF-α and IL-6 mRNA abundance. The above results demonstrate that single and combined treatments with DON and PAT triggered inflammatory responses in the liver. Among all treatment regimens, the combined administration of DON and PAT exerted the most potent regulatory effects on the mRNA levels of MyD88, IFN-γ, and JAK2.

### 2.5. Effects of Individual or Combined Treatment with DON and PAT on Hepatic Oxidative Stress in Mice

**Figure 5:**
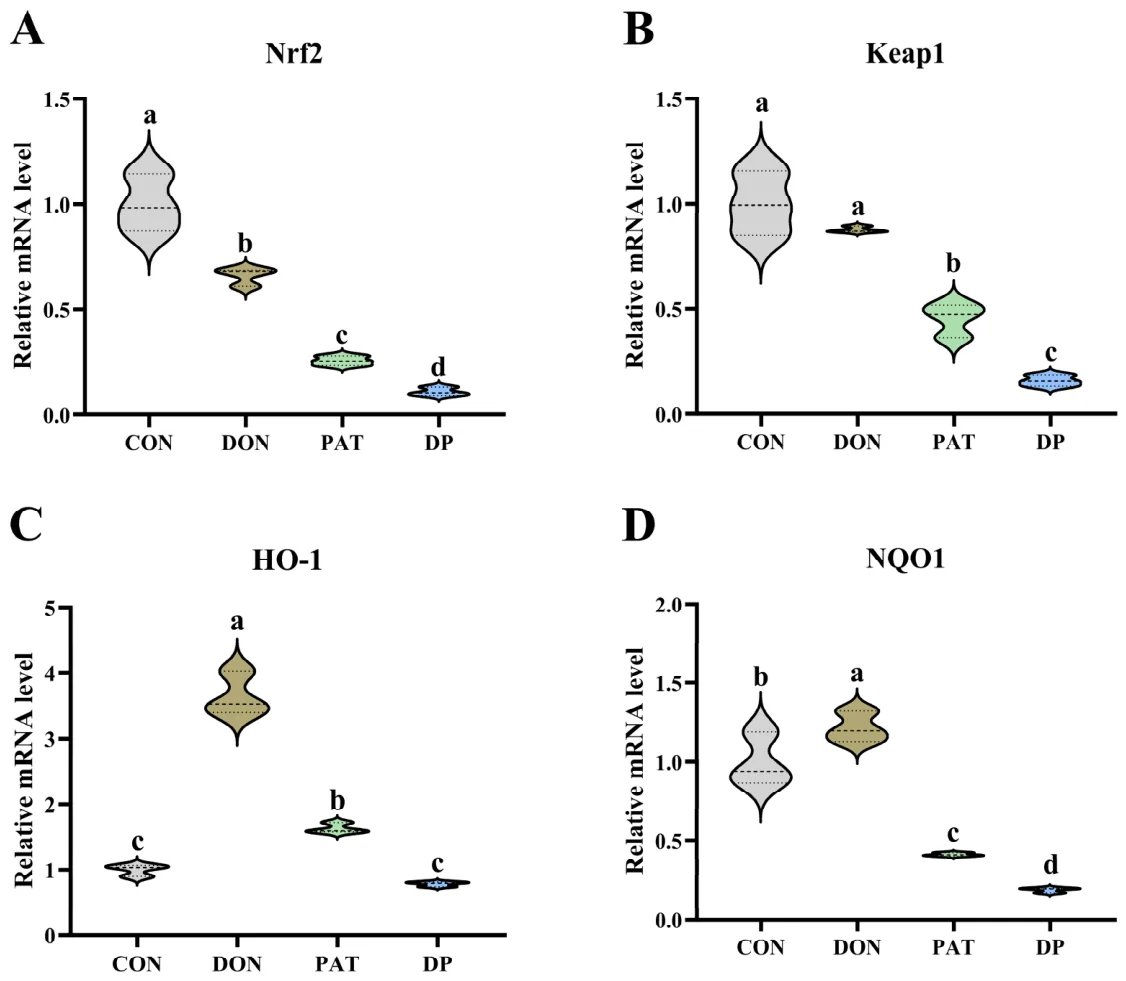
Individual and combined effects of DON and PAT on liver oxidative stress in mice (*n* = 3). Note: (**A**–**D**) represent the mRNA abundances of Nrf2, Keap1, HO-1, and NQO1 in mouse liver, respectively. Identical lowercase superscript letters (e.g., a, b, c) indicate no significant differences among treatment groups (*p* > 0.05); different lowercase superscript letters indicate significant differences (*p* < 0.05).

Figure 5 shows alterations in the expressions of oxidative stress-related genes in the liver of mice exposed to DON, PAT, or DP. The results showed that the mRNA level of Nrf2 in the DON group was significantly reduced (*p* < 0.05), while the mRNA levels of heme oxygenase (HO-1) and NQO1 in the DON group were significantly elevated (*p* < 0.05), compared with the CON group. No significant change was observed in the mRNA level of Keap1 (*p* > 0.05) between the CON and DON groups. The mRNA levels of Nrf2, Keap1, and NQO1 in the PAT group were significantly reduced, whereas the mRNA level of HO-1 was significantly elevated (*p* < 0.05), compared with the CON group. The mRNA levels of Nrf2, Keap1, and NQO1 in the DP group were significantly lower than those in the other three groups (*p* < 0.05). There was no significant difference in the mRNA level of HO-1 between the CON and DP groups. Collectively, single DON or PAT exposure induced hepatic oxidative stress in mice, and their combined exposure exacerbated such oxidative stress to produce enhanced toxic effects.

### 2.6. RNA-Seq Analysis

**Figure 6:**
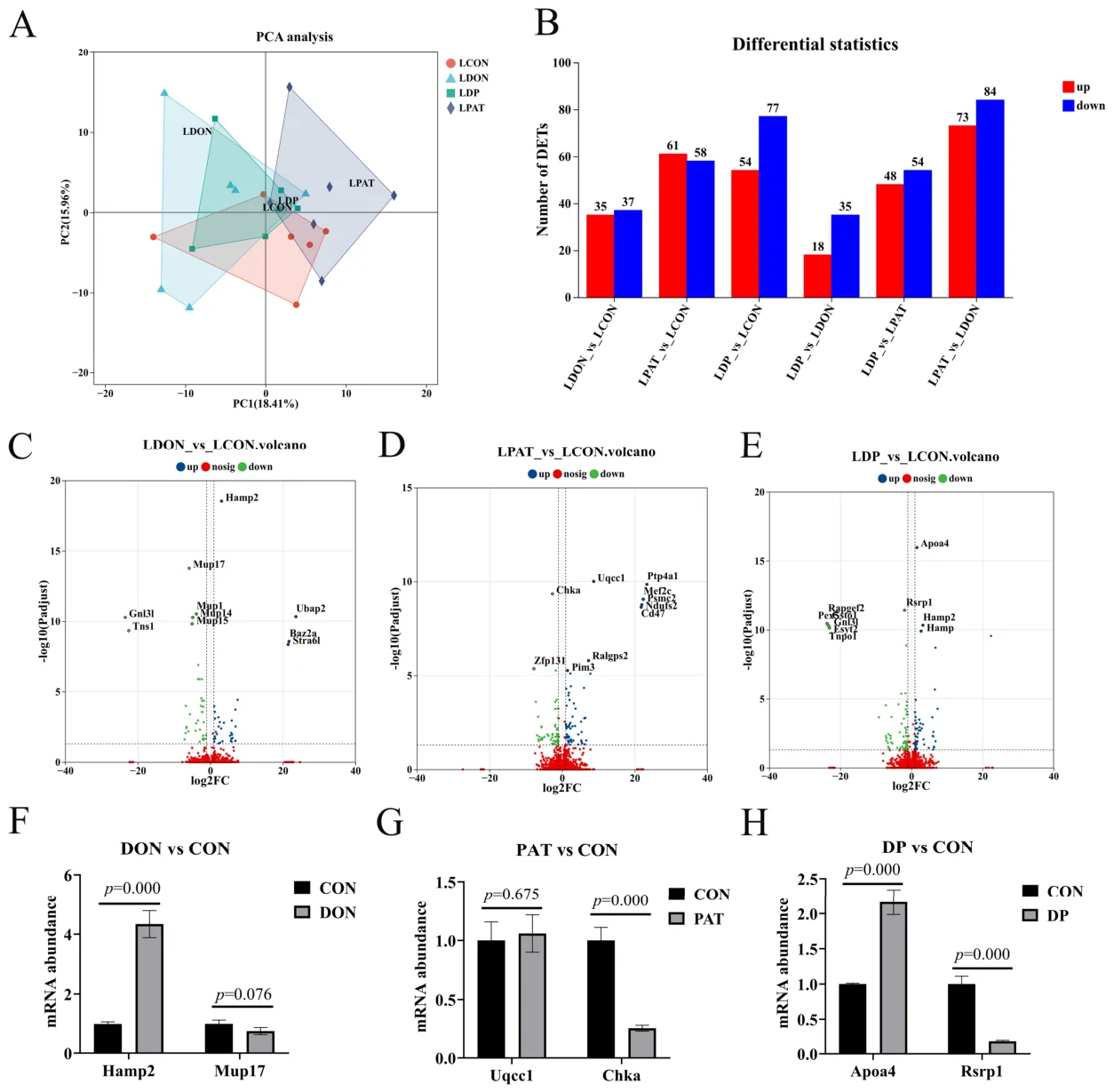
Individual and combined effects of DON and PAT treatments on liver gene transcription levels in mice (*n* = 6). (**A**) PCA plots of samples in all groups. (**B**) Statistical charts of DEGs between two groups. (**C**–**E**) Volcano plots of upregulated and downregulated DEGs in the DON, PAT, and DP groups vs. the CON group. (**F**–**H**) The mRNA abundance of the most significantly upregulated and downregulated differentially expressed genes (DEGs) in the DON, PAT, and DP groups relative to the CON group, respectively.

Transcriptomic analysis was performed to further investigate the potential mechanisms underlying the hepatic toxicity induced by individual and combined exposure to DON and PAT in mice, and to examine the alterations in gene expression in mouse liver tissue. Principal component analysis (PCA) was conducted based on gene expression levels (Figure 6A). The results showed that 18.41% and 15.96% of the total variation were explained by principal component 1 and principal component 2, respectively. Differentially expressed genes with |log2(fold change)| > 1 and adjusted *p*-value < 0.05 were identified through pairwise comparisons between any two of the four groups (Figure 6B). 35, 61, and 54 upregulated DEGs as well as 37, 58, and 77 downregulated DEGs were detected in the DON, PAT, and DP groups relative to the CON group, respectively. In detail, 18 upregulated DEGs and 35 downregulated DEGs were recognized in the comparisons between the DP and DON groups, and 48 upregulated DEGs and 54 downregulated DEGs were recognized in the comparisons between the DP and PAT groups. When the PAT group was compared with the DON group, 73 upregulated DEGs and 84 downregulated DEGs were found. The most significantly upregulated and downregulated DEGs were Hamp2 and Mup17 in the DON group (Figure 6C), Uqcc1 and Chka in the PAT group (Figure 6D), and Apoa4 and Rsrp1 in the DP group (Figure 6E), respectively, compared with the CON group. The relative mRNA abundance of these differentially expressed genes was further verified. According to Figure 6F–H, Hamp2 (DON group), Chka (PAT group), Apoa4 and Rsrp1 (DP group) displayed remarkably altered mRNA levels compared with the control group, matching the DEG regulation trends in Figure 6C–E. However, Mup17 in the DON group and Uqcc1 in the PAT group showed non-significant expression changes versus the control group, conflicting with the trends shown in Figure 6C,D.

**Figure 7:**
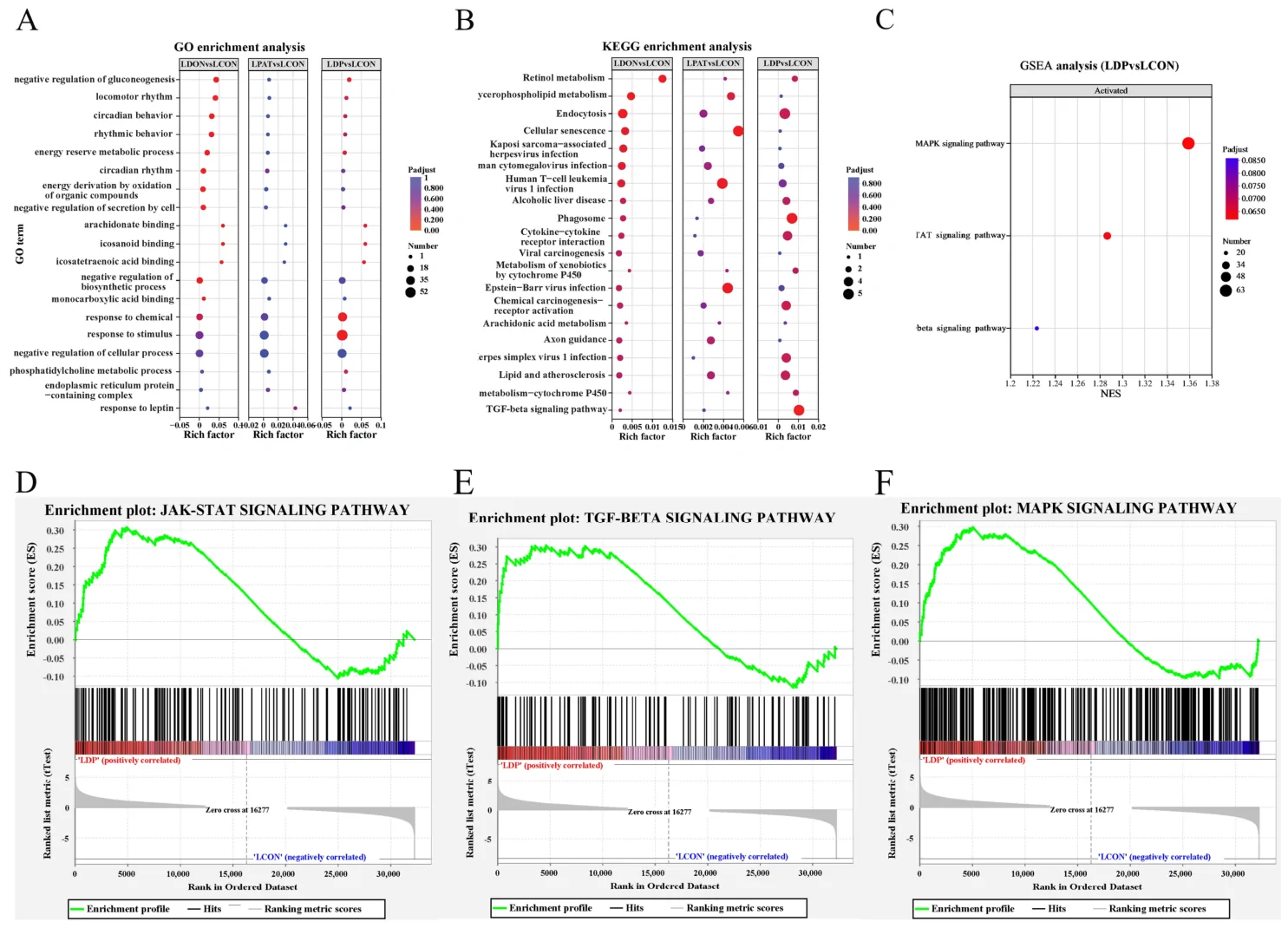
GO enrichment and KEGG pathway analysis of hepatic gene transcription in mice upon individual and combined exposure to DON and PAT. (*n* = 6). (**A**) GO enrichment analysis plots of DEGs in DON, PAT and DP groups vs. the CON group. (**B**) KEGG enrichment analysis plots of DEGs in the DON, PAT and DP groups vs. the CON group. (**C**) Normalized gene set enrichment analysis (GSEA) plots. (**D**–**F**) Enrichment analysis plots of genes upregulated in the MAPK, JAK-STAT and TGF-β signaling pathways in the DP group.

DEG sets with a 2-fold change were separately established for DON vs. CON, PAT vs. CON, and DP vs. CON comparisons, and GO enrichment analysis was subsequently performed (Figure 7A). The results revealed that the DEG sets in the three groups were most significantly enriched in biological processes including response to chemical substance, response to stimulus, and the negative regulation of cellular process. The highest DEG enrichment was observed in the DP vs. CON comparison, indicating that combined treatment with DON and PAT triggered alterations in the expression of more genes. Subsequently, KEGG enrichment analysis was conducted on the three DEG sets. The results demonstrated that these DEGs were mainly enriched in the MAPK signaling pathway, JAK-STAT signaling pathway, TGF-β signaling pathway, AMPK signaling pathway, cGMP-PKG signaling pathway, and other pathways (Figure 7B). Furthermore, DEGs enriched in the MAPK, JAK-STAT, and TGF-β signaling pathways in the DP vs. CON comparison were analyzed (Figure 7C–F). In summary, combined exposure to DON and PAT may trigger activation of the MAPK, JAK-STAT, and TGF-β signaling pathways.

## 3. Discussion

Mycotoxins are recognized as contaminants that impose detrimental impacts on the health of humans and animals. Previous studies revealed both DON and PAT as foodborne intestinal toxins [25]. Intestinal villi and intestinal tight junctions were destroyed by DON and PAT exposure; mucus secretions and inflammatory responses were reduced, oxidative stress was reduced, and gut microbiota was disturbed. In addition, metabolic imbalance was triggered, and the digestion and absorption of nutrients were impaired, which finally resulted in retarded body weight gain in animals [26,27,28,29,30,31,32]. In the present study, body weight gain in mice was suppressed by treatment with DON and PAT alone or their combination, and a stronger inhibitory effect was detected in the combined treatment group. Thus, the negative effects of DON and PAT on animal growth were further verified. Under physiological homeostasis, the hepatic organ index of mice remains stable, and alterations in the hepatic organ index can serve as an intuitive macroscopic biomarker to comprehensively evaluate the severity of hepatic lesions [33]. DON induces mitochondrial vacuolization and endoplasmic reticulum stress, thereby triggering ultrastructural damage in hepatocytes; meanwhile, DON activates ferroptosis, oxidative stress and inflammatory responses, resulting in lipid metabolism disorders, inflammatory cellular infiltration, nuclear rupture, and karyolysis, ultimately inducing hepatomegaly and increasing the hepatic organ index [34,35,36,37,38]. PAT also activates oxidative stress, pyroptosis, and inflammatory responses in hepatocytes, inducing hepatic inflammatory infiltration, karyolysis, and hepatocyte injury, accompanied by decreased hepatic organ index and metabolic dysfunction [17,23,39]. In the present study, single DON exposure elevated the hepatic organ index and induced hepatomegaly and injury in mice, while single PAT exposure reduced the hepatic organ index and triggered hepatic atrophy and damage. Combined exposure to DON and PAT exerted mutual antagonism, restoring the hepatic organ index to the level of the normal control group. The aforementioned histomorphological lesions were observed in the livers of mice subjected to individual DON or PAT treatment, and microscopic injuries including inflammatory infiltration, karyolysis, and organelle vacuolization were still detectable in the combined DON and PAT treatment group. These findings demonstrate that the two toxins exert an antagonistic effect at the macroscopic level of hepatic injury, while toxic interactions at the microscopic molecular level cannot be excluded.

Numerous unique macrophage populations were detected in the liver. These cells were involved in the recognition and clearance of pathogens and toxic substances, and pro-inflammatory and anti-inflammatory cytokines were secreted to regulate inflammatory responses [40]. TLR4/MyD88/NF-κB signaling pathway is recognized as a canonical pathway regulating inflammatory responses. Exogenous danger signals are recognized by the TLR4 receptor, and MyD88 is subsequently recruited intracellularly. A series of kinase reactions (including IRAK, TRAF6, TAK1, and others) is initiated, and signal transduction complexes are formed. The IKK complex is eventually activated, and the IκBα protein is degraded. After degradation, NF-κB is released and translocated into the nucleus, the transcription of relevant genes is initiated, pro-inflammatory cytokines (e.g., TNF-α, IL-6, IL-1β) are produced, and inflammatory responses are thereby triggered [41]. In the present study, the mRNA levels of TLR4, IκBα, IL-1β, and STAT3 were significantly elevated in the DON group, while no significant changes were observed in the expression of MyD88, IL-6, and other related genes. In contrast, the level of TNF-α mRNA was remarkably downregulated. The results indicate that hepatic inflammation induced by DON is mainly mediated through the TLR4/IκBα/STAT3 pathway, rather than the complete MyD88-dependent pathway. TLR4-mediated inflammatory responses in intestinal and hepatic tissues were reported to be activated by DON [42,43]. The downregulation of TNF-α observed in this study was suggested to be associated with tissue-specific regulation induced by DON or compensatory feedback inhibition caused by the excessive activation of other inflammatory mediators. The mRNA levels of MyD88, IκBα, IL-1β, IL-6, and TNF-α were significantly elevated in the PAT group, whereas no significant alterations were found in the expressions of TLR4 and other related genes. It was demonstrated that hepatic inflammation induced by PAT was dependent on the MyD88/NF-κB pathway, and the upregulation of TLR4 was not involved in this process. These results are consistent with previous reports that inflammatory responses in multiple cell types were induced by PAT by activating the NF-κB signaling pathway [30,31]. The nuclear translocation of NF-κB might be promoted by PAT through specific binding with MyD88, inducing the expression of pro-inflammatory factors. Notably, seven inflammation-related genes (including TLR4, MyD88, IL-1β, and others) were significantly upregulated in the DP group, and IκBα and STAT3 mRNA levels were not significantly changed. The activation effects on hepatic inflammatory pathways were achieved by combined treatment with DON and PAT. The upregulation amplitudes of MyD88, IFN-γ, and JAK2 in the DP group were significantly higher than those in the single-treatment groups. These results indicate that the interplay between TLR4 and MyD88-dependent pathways drives the sustained activation of hepatic inflammatory pathways and the amplification of inflammatory cascades, corroborating previous reports of their co-regulation in inflammatory responses [44].

Cellular apoptosis is regulated by the Bcl-2 family and the caspase pathway, and the Bax/Bcl-2 ratio and Caspase-3 are key molecules involved in apoptosis [45]. In the present study, the mRNA levels of Bax and Caspase-3 and the Bax/Bcl-2 ratio were significantly upregulated in the DON group, while the mRNA level of Bcl-2 was markedly downregulated. The severity of hepatocyte apoptosis was significantly higher in the DON group than that in the PAT alone and combined treatment groups. These results are consistent with a previous study that the mitochondrial apoptotic pathway in hepatocytes was triggered by DON by the upregulation of Bax and downregulation of Bcl-2 [46]. The significant activation of Caspase-3 was detected, which further confirmed that apoptosis induced by DON was dependent on the canonical caspase cascade. In the PAT group, only the expression of Bcl-2 was downregulated, and the Bax/Bcl-2 ratio was elevated, whereas no significant changes were observed in the expressions of Bax and Caspase-3. These findings indicate that the cell apoptosis induced by PAT was mild and was mediated by an imbalance in the Bcl-2 family. The changes in apoptosis-related indices were weaker in the DP group than those in the DON group. Such results are speculated to be related to the antagonistic effect of PAT on the activation of Caspase-3 or inflammation-mediated anti-apoptotic feedback. The interactive regulation among toxins was reported in the previous study focused on combined exposure to multiple toxicants [47].

The Nrf2-Keap1 pathway is recognized as the core antioxidant defense pathway in organisms, and the expression of downstream antioxidant genes including HO-1 and NQO1 is regulated by Nrf2 [48]. Nrf2 expression was decreased after DON treatment, while HO-1 and NQO1 expressions were significantly increased in this study. These observations suggested that compensatory antioxidant responses were activated in hepatocytes, and a stress threshold effect of Nrf2 was confirmed [49,50]. The Nrf2-Keap1 pathway was directly suppressed by PAT, and the antioxidant capacity of the body was impaired. The upregulation of HO-1 is regarded as an acute stress response. Thus, our results indicate that different mechanisms were utilized by DON and PAT to induce oxidative stress. The expression of Nrf2 and its downstream antioxidant genes was comprehensively downregulated after combined treatment, and hepatic oxidative stress was further aggravated. These results are consistent with the synergistic oxidative damage caused by combined exposure to multiple mycotoxins [51].

Partial separation was only observed in the PAT group in transcriptomic PCA, while prominent overlap existed among the CON, DON, and DP groups. These results suggested that DON and PAT single or combined treatments exerted differentiated effects on hepatic gene expression profiles in mice. According to the DEG statistical results, the most upregulated DEGs (79) were detected in the PAT group compared with the control group, while a greater number of downregulated DEGs (48) was identified in the DP group than in the DON or PAT group. Moreover, a unique expression pattern of DEGs was observed between the DP group and the DON or PAT group, suggesting that toxic effects were amplified by combined treatment through novel gene regulatory networks. Hepatic Chka, Hamp2, Apoa4, and Rsrp1 participate in the modulation of metabolism, inflammation, and oxidative stress [52,53,54,55]. Transcriptome analysis and RT-qPCR validation further prove that DON, PAT, and DP co-exposure induce differentiated liver toxicity. Hepatic dysfunction was further aggravated by the abnormal expression of these genes. GO enrichment analysis showed that DEGs in all three treatment groups were highly enriched in biological processes including response to chemical substance, response to stimulus, and negative regulation of cellular processes. The highest enrichment intensity was observed in the DP group, which was consistent with the synergistic toxicity phenotype induced by the combined treatment. The results indicate that broader cellular stress response mechanisms were activated by combined exposure. Kyoto Encyclopedia of Genes and Genomes (KEGG) enrichment analysis further confirmed that the identified DEGs were mainly distributed in the MAPK, JAK-STAT, and TGF-β signaling pathways. The largest number of DEGs was enriched in the MAPK signaling pathway, suggesting that this pathway is the central regulatory hub for combined toxicity. The MAPK signaling pathway was recognized as a critical pathway mediating cellular stress, inflammation, and apoptosis. The expressions of pro-inflammatory cytokines (IL-1β and IL-6) and apoptosis-related genes (Bax and Caspase-3) were regulated by MAPK signaling pathway activation via the phosphorylation of downstream transcription factors [56], which is highly consistent with the phenotypes of enhanced inflammation and activated apoptosis in the combined treatment group. The enrichment of the JAK-STAT signaling pathway verified the previous molecular findings that hepatic inflammation induced by combined treatment of DON and PAT was mediated by this pathway. A previous study reported that the release of inflammatory cytokines was enhanced and hepatic injury was aggravated by mycotoxins by activating the JAK-STAT signaling pathway [57]. The activation of the TGF-β signaling pathway suggests that long-term toxic effects, including hepatic fibrosis, were involved in the combined treatment [58]. Hepatic injury was further amplified by crosstalk among the TGF-β, MAPK, and JAK-STAT signaling pathways.

## 4. Conclusions

Exposure to DON or PAT alone suppressed growth in mice and elicited hepatic pathological damage, inflammation, apoptosis of hepatocytes, and oxidative stress; DON proved more potent than PAT in inducing hepatocyte apoptosis. In contrast, combined exposure to the two mycotoxins perturbed the hepatic transcriptome, triggered a cascade of signaling pathways centered on MAPK, and exacerbated liver injury.

## 5. Materials and Methods

### 5.1. Experimental Materials and Animal Treatment

Twenty-four specific pathogen-free male C57BL/6 mice at about 6 weeks of age were purchased from Henan Chunying Biotechnology Co., Ltd. (Zhengzhou, China). They were randomly assigned to 4 groups, with 6 replicates in each group (1 mouse per cage with dimensions of 320 × 21 × 170 mm). The mice were intragastrically administered with DON, PAT, DON + PAT (DP), or without DON and PAT (the control group, CON) for 28 days, respectively. The mice were maintained in an environment at 25 ± 2 °C and 65–70% humidity with 12 h light and 12 h darkness. The mice were allowed to acclimatize for 7 days; food and water were given ad libitum.

DON (HY-N6684, purity ≥ 99.8%) and PAT (B24209, purity ≥ 99%) were purchased from MedChemExpress (South Brunswick, NJ, USA) and Shanghai Yuanye Bio-Technology Co., Ltd. (Shanghai, China), respectively. The mice in the CON group received intragastric administration of normal saline. Mice in the DON, PAT, and DP groups were intragastrically treated daily with DON (1.5 mg/kg BW), PAT (2.0 mg/kg BW), and the combination of DON (1.5 mg/kg BW) plus PAT (2.0 mg/kg BW), respectively. The selected mycotoxin doses were established according to prior reports [16,17,19,36,59] and findings from our preliminary trials. All intragastric administrations were performed at a volume of 0.2 mL per mouse per day. After 28 consecutive days of administration, the mice were fasted, and their live body weights were recorded as W0. Subsequently, the mice were euthanized, and liver tissues were excised and weighed as W1. The hepatic index was calculated according to the formula: Hepatic index = (W1/W0) × 100%. Liver samples were then divided into two aliquots. One part was immediately fixed in 4% paraformaldehyde for histopathological evaluation, and another part was placed in liquid nitrogen for subsequent analyses of quantitative real-time polymerase chain reaction (PCR) and RNA-sequencing (RNA-seq). All the animal experiments have been approved by the Ethics Committee of Henan Agricultural University (Approval No.: HNND2025082508), which were conducted strictly following the relevant guidelines.

### 5.2. Histopathological Examination

Liver tissues fixed in formalin solution underwent standard processing including dehydration, embedding, sectioning, dewaxing, staining, and mounting. Whole-slide digital images were acquired using a digital slide scanner (KF-PRO-400, KF Bio, Seoul, Republic of Korea). Four-μm-thick paraffin-embedded tissue sections were dewaxed in xylene I and II for 5–10 min, respectively, followed by rehydration in a graded ethanol series (100%, 95%, 90%, 80%, and 70% ethanol) for 1–2 min per step, with a final rinse in distilled water. Thereafter, antigen retrieval was performed by immersing the sections in 0.01 M sodium citrate buffer (pH = 6.0) to unmask antigenic epitopes. After cooling to room temperature, primary antibody working solutions (antibodies against GAPDH, IL-6, and TNF-α were obtained from MedChemExpress) diluted to 1:200 were added to cover the tissue sections uniformly, followed by incubation in a constant-temperature incubator at 37 °C for 60 min. Subsequently, the sections were washed with phosphate-buffered saline (PBS) three times (5 min per time) to eliminate unbound primary antibodies. The corresponding horseradish peroxidase-conjugated goat anti-rabbit secondary antibodies (purchased from MedChemExpress) were then applied, and incubation was continued at 37 °C for 30 min to amplify the staining signal. The 3,3′-diaminobenzidine chromogenic kit was utilized for color development, and the reaction was stopped once distinct brown specific staining was visualized. The sections were counterstained with hematoxylin for 5–10 min to label cell nuclei, and finally subjected to routine dehydration, clearing, and mounting with neutral balsam.

### 5.3. qRT-PCR Assay

A qRT-PCR assay was performed as previously described [60] and detailed in the Supplementary Materials. All primers used for gene amplification were obtained from Sangon Biotech Co., Ltd. (Shanghai, China), and their sequences were presented in Supplementary Table S1. The relative mRNA expression levels of target genes were quantified via the classic 2^−ΔΔCt^ calculation method. GAPDH was selected as the housekeeping reference gene for internal normalization. Each biological sample was set with three independent technical replicates during PCR amplification.

### 5.4. RNA-Sequencing Analysis

Total RNA was extracted from mouse liver, and then its quantity, purity, and integrity were determined. Subsequently, RNA was randomly interrupted, reverse transcribed into cDNA, and sequenced on an Illumina NovaSeq X Plus (Shanghai Majorbio Co., Ltd., Beijing, China). The expression levels of genes were calculated using the fragments per kilobase of transcripts per million fragments mapped (TPM) method. Differentially expressed genes (DEGs) were judged using the DESeq method, with an absolute value of |log2(fold change)| > 1 and adjusted *p*-value < 0.05. Then, Gene Ontology (GO, http://geneontology.org/ (accessed on 26 June 2026)), Kyoto Encyclopedia of Genes and Genomes (KEGG, http://www.genome.jp/kegg/ (accessed on 26 June 2026)), and Gene Set Enrichment Analysis (GSEA, https://www.gsea-msigdb.org/ (accessed on 26 June 2026)) were performed for functional annotation, pathway enrichment analysis, and gene enrichment analysis of the DEGs. Enrichment analysis was conducted using clusterProfiler, and significantly enriched biological processes and pathways (*p* < 0.05) were screened to reveal the biological processes, molecular functions, and signaling pathways associated with the DEGs.

### 5.5. Statistical Analysis

Normality of continuous variables was assessed via the Shapiro-Wilk test, and homogeneity of variances among groups was verified by Bartlett’s test. All datasets satisfied both normality and homoscedasticity assumptions; accordingly, one-way analysis of variance (ANOVA) was performed for overall intergroup comparisons, followed by Tukey’s test for pairwise comparisons. Statistical significance was set at *p* < 0.05. All graphs were plotted using GraphPad Prism 10 (GraphPad Software, Boston, MA, USA).

## Data Availability

The data presented in this study are openly available in NCBI at https://dataview.ncbi.nlm.nih.gov/object/PRJNA1460996 (accessed on 18 August 2026), reference number PRJNA1460996.

## Supplementary Materials

The following supporting information can be downloaded at: https://www.mdpi.com/article/10.3390/toxins18080355/s1, Table S1: Primer sequence information.
