# Supplementary material for: Combined Exposure to Deoxynivalenol and Patulin Aggravates Liver Injury in Mice via Triggering Inflammation, Apoptosis, and Oxidative Stress
Source: Toxins (Basel). 2026 Aug 20;18(8):355. doi: 10.3390/toxins18080355 (PMC13517715; doi:10.3390/toxins18080355)
Supplement: Supplementary file 1 [file toxins-18-00355-s001.zip › toxins-4433376-supplementary.pdf]

Supplementary Materials: Combined Exposure to Deoxynivalenol and Patulin Aggravates Liver Injury in Mice via Triggering Inflammation, Apoptosis, and Oxidative Stress

Qingqing Zhao, Zenghao Xu, Xianglong Dai, Xingyu Zhang, Maolong Li, Juan Chang, Qingqiang Yin, Guoyu Yang and Chaoqi Liu

Table S1. Primer sequence information.

Gene	Primer sequences (5'-3')	Accession NO.	Product size (bp)
GAPDH	F: CAGGAGAGTGTTTCCTCGTCC<br>R: GATGGGCTTCCCGTTGATGA	NM_001289726.2	247
Bax	F: TGAAGACAGGGGCCTTTTTG<br>R: AATTCGCCGGAGACACTCG	NM_007527.4	140
Bcl-2	F: GTCGCTACCGTCGTGACTTC<br>R: CAGACATGCACCTACCCAGC	NM_177410.3	284
Caspase3	F: GGGGAGCTTGGAACGCTAAG<br>R: CCGTACCAGAGCGAGATGAC	NM_009810.3	232
TLR4	F: CTGGCTGGTTTACACGTCCA<br>R: CCTGCCAGAGACATTGCAGA	NM_021297.3	200
MyD88	F: GCGCTTAACGTGGGAGTG<br>R: ATATCCTCACGGTCTAACAAGG	NM_010851.3	233
IκBα	F: AGACCTGGCCTTCCTCAACTTCC<br>R: CTGCGTCAAGACTGCTACACTGG	NM_010907.2	199
IL-1β	F: TGCCACCTTTTGACAGTGATG<br>R: AAGGTCCACGGGAAAGACAC	NM_008361.4	220
IL-6	F: CAACGATGATGCACTTGCAGA<br>R: TGTGACTCCAGCTTATCTCTTGG	NM_001314054.1	251
TNF-α	F: ACAAGGTACAACCCATCGGC<br>R: GCTACGACGTGGGCTACAG	NM_013693.3	133
IFN-γ	F: CGGCACAGTCATTGAAAGCC<br>R: TGCATCCTTTTTCGCCTTGC	NM_008337.4	268
JAK2	F: GAGGTGGTCGCTGTGAAGAA<br>R: GCACTGTAGCACACTCCCTT	NM_001048177.3	131
STAT3	F: CAAGGGCTTCTCCTTCTGGG<br>R: GGGGGCTTTGTGCTTAGGAT	NM_011486.6	141
Nrf2	F: AAGCACAGCCAGCACATTCTCC<br>R: TGACCAGGACTCACGGGAACTTC	NM_010902.5	130
Keap1	F: TGGTCGCCCTGTGCCTCTATG<br>R: ATGCCACTCGTCCCGCTCTG	NM_001110307.1	147
HO-1	F: ACCGCCTTCCTGCTCAACATTG<br>R: CTCTGACGAAGTGACGCCATCTG	NM_010442.2	104
NQO1	F: CCATGTACGACAACGGTCCT<br>R: ACGCAGGATGCCACTCTGAA	NM_008706.5	143
Apoa4	F: CCGTTTCTTCTGACTCCGGG<br>R: GTCCTGGAAGAGGGTACTGAG	NM_007468.2	260
Chka	F: CAGTGTCATCAGGGGTGGTC<br>R: TGAGCTTGTTCGGATCCCTC	NM_001271496.2	159
Hamp2	F: CACCTATCTCCAGCAACAGATGA<br>R: ACAGATACCACAGGAGGGTTTG	NM_183257.5	172
Mup17	F: AAGATGCTGCTGCTGCTGT<br>R: GGAGCACTCTTCATCTCTTACAGT	NM_001200006.1	246
Uqcc1	F: GTGCGAGTCCTTAGGAACCA<br>R: AGGCTGTGGGAAATCTTGGG	NM_001355441.1	234
Rsrp1	F: GCCAAAGGTGGAGCATTCAG<br>R: AAGTGCAGTTTTGGCAACCC	NM_023665.3	251
